# Assessment of Explicit Representation of Dynamic Viral Processes in Regional Marine Ecological Models

**DOI:** 10.3390/v14071448

**Published:** 2022-06-30

**Authors:** Le Xie, Rui Zhang, Ya-Wei Luo

**Affiliations:** State Key Laboratory of Marine Environmental Science, College of Ocean and Earth Sciences, Xiamen University, Xiamen 361102, China; xiele@stu.xmu.edu.cn (L.X.); ruizhang@xmu.edu.cn (R.Z.)

**Keywords:** marine virus, heterotrophic bacteria, marine ecological model, data assimilation

## Abstract

Viruses, the most abundant microorganisms in the ocean, play important roles in marine ecosystems, mainly by killing their hosts and contributing to nutrient recycling. However, in models simulating ecosystems in real marine environments, the virus-mediated mortality (VMM) rates of their hosts are implicitly represented by constant parameters, thus ignoring the dynamics caused by interactions between viruses and hosts. Here, we construct a model explicitly representing marine viruses and the VMM rates of major hosts, heterotrophic bacteria, and apply it to two sites in the oligotrophic North Pacific and the more productive Arabian Sea. The impacts of the viral processes were assessed by comparing model results with the viral processes enabled and disabled. For reliable assessments, a data assimilation method was used to objectively optimize the model parameters in each run. The model generated spatiotemporally variable VMM rates, generally decreasing in the subsurface but increasing at the surface. Although the dynamics introduced by viruses could be partly stabilized by the ecosystems, they still caused substantial changes to the bacterial abundance, primary production and carbon export, with the changes greater at the more productive site. Our modeling experiments reveal the importance of explicitly simulating dynamic viral processes in marine ecological models.

## 1. Introduction

Marine viruses are the most abundant biological entities and the greatest reservoir of genetic diversity in the world’s oceans [1,2,3]. They are estimated to, in every second, infect 10^23^ marine organisms, most of which are bacteria [4,5], and kill 10–40% of bacterioplankton per day, a rate comparable to those grazed by zooplankton [2,6,7]. Viruses are, therefore, one of the key players in marine ecosystems, particularly in the microbial loop [8,9,10]. One of the important roles that viruses have in regulating the diversity of the bacterial community is to kill the bacterial groups that are the most competitive in acquiring resources, a model named “kill-the-winner” [11]. The viral lysis of the infected bacteria results in a release of organic matter in both dissolved and particular forms, a large fraction of which is consumed and respired by heterotrophic bacteria and phytoplankton and further recycled to inorganic nutrients to support primary production [12,13]. This process also increases community respiration and decreases the transfer of photosynthetically fixed carbon to higher trophic levels [14].

Despite their importance, virus-related processes have not been represented and simulated in most marine ecological models. With the increasing understanding of marine viruses and their ecological roles, a limited number of trials modeling marine viral processes have been conducted since the 1990s, describing viral infection, the latent period and lysis [15,16,17,18,19,20,21]. A pioneering theoretical model involving marine viral processes separated phytoplankton into a susceptible group and a virally infected group, in which the infection rate depended on the contact rate between the two phytoplankton groups while the viruses themselves were not simulated [15]. Afterward, explicit representations of marine viruses were added in several models [17,21,22,23,24,25], in which, however, viruses were, practically, only diagnostic variables that were calculated from the abundance of their host bacteria by using constant virus-to-host ratios.

More importantly, these models set constant virus-mediated mortality (VMM) rates of bacteria. These models, therefore, were largely similar to previous non-virus models in simulating the effects of viruses on other components of marine ecosystems, considering that most non-virus models also assigned constant mortality rates to the simulated bacteria. The viral infection rates of hosts, and subsequently the VMM rates, are highly variable [26,27,28,29,30,31,32,33,34,35] and can be impacted by the abundance of viruses and hosts, the temperature and nutrients [36]. As a first-order estimation, the rate of viral infection depends on the contact rate between the viruses and hosts [37] and thus can be assumed to be proportional to the product of viral and host abundances [38]. The parameterization of this assumption has been implemented in several theoretical models, resulting in the dynamic simulation of the VMM [16,18,19,39,40,41,42]. It can be interesting to test how an explicit representation of viral processes, in particular, the simulation of spatiotemporally variable VMM rates, can change the performance of marine ecological models driven by realistic dynamic conditions. Therefore, the importance and necessity of explicit representations of viral processes in ecological models can be evaluated and compared in ocean regions with different biogeochemical characteristics.

In this study, we modified an existing marine ecological model [43] by adding a viral module in which viruses and their related processes were explicitly represented. The realistic dynamic oceanic conditions in the model were simulated by a physical model at specific locations. Our model in this study was not applied to the global ocean but to two contrasting regional sites in the oligotrophic North Pacific subtropical gyre and in the Arabian Sea, which was more productive. We evaluated the model performance by comparing the simulated results of host abundance and mortality, primary production and carbon export in model cases with and without the viral module. To avoid the influence of artificially selected parameter values on the objective evaluation of the model performance, a data-assimilation approach was used to optimize the model parameters in each case, to minimize the differences between the observations and model outputs.

## 2. Materials and Methods

### 2.1. Study Sites

The model was applied to the site of the Hawaii Ocean Time-series (HOT) program (Station ALOHA, 22.75° N, 158.00° W) (hereafter referred to as “HOT”) and an offshore site S7 in the Arabian Sea (16.0° N, 62.0° E) (hereafter referred to as “AS”). The HOT site was established in 1988 and is located within the southward backflow of the eastern North Pacific Subtropical Gyre [44]. The site is generally depleted of nutrients. The seasonal variability of primary production and other biogeochemical characteristics is relatively weak at HOT. According to the data availability, we ran our model for 2002 at HOT. The Arabian Sea is subject to seasonal monsoons, which result in different microbial dynamics compared to HOT. The model was run for 1995 at AS due to data availability, and the two N_2_-fixing groups were not simulated, considering that N_2_ fixation was presumably relatively weak at this site [43]. The simulation and comparison at these two sites with contrasting biogeochemical characteristics were undertaken to provide more information on the performance of the viral module.

### 2.2. Standard Model

**Figure 1:**
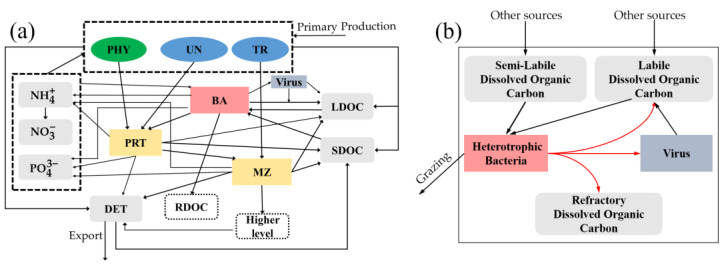
Model structure. (**a**) Diagram of the model structure showing stocks and flows of state variables. Dashed borders for “higher levels” and “refractory DOC” indicate they were not modeled explicitly in this study. Several state variables are grouped by dashed rectangles. A flow arrow ending on a grouping rectangle means the flow applies to all the state variables inside the rectangle. Unicellular diazotrophs and *Trichodesmium* are disabled at the AS (Arabian Sea) site. See main texts for the acronyms. (**b**) Structure zoomed in to the viral module. The viral lysis of heterotrophic bacteria leads to the release of labile and refractory DOC and viruses.

Our viral module was incorporated into an existing biogeochemical model [43], which is hereafter referred to as the “standard model”. Briefly, the standard model simulated stocks and flows of carbon (C), nitrogen (N) and phosphorus (P) in non-diazotrophic phytoplankton (PHY), N_2_-fixing unicellular diazotroph (UN) and *Trichodesmium* spp. (TR), heterotrophic bacteria (BA), protozoan zooplankton (PRT), metazoan zooplankton (MZ), labile and semi-labile dissolved organic carbon (LDOC and SDOC), sinking detritus (DET), and inorganic nutrients including nitrate (NO_3_^−^), ammonium (NH_4_^+^) and phosphate (PO_4_^3−^) (Figure 1a). The production of refractory dissolved organic carbon (RDOC) and grazing of metazoan zooplankton by higher trophic levels were implicitly represented as model closure terms. In this study, we focused on the modeled carbon stocks and flows. More details of the standard model are described in Appendix A.

The model was a one-dimensional vertical model with multiple layers for the upper ocean. For AS, the model had 20 layers, including 10 surface layers of 5 m each and 10 layers of 10 m each below, covering the upper water column of 150 m. For HOT, an additional five layers of 10 m were added at the bottom because of its deeper euphotic zone, therefore covering the upper 200 m. The model was forced by photosynthetically active radiation (PAR), temperature, mixed layer depth (MLD), vertical velocity and vertical diffusivity. A 90-day spin-up run was conducted before the simulations of the study period to avoid substantial influence from the initial conditions of the state variables.

The model parameters were objectively optimized to minimize the differences between model outputs and observations of multiple variables (Table 1) by utilizing a data-assimilation method with a variational adjoint scheme [45]. The observational data for Station ALOHA (Supplementary Table S2) were obtained at http://hahana.soest.hawaii.edu/hot/hot-dogs/interface.html (accessed 30 June 2022), except for bacterial production data, which were measured during HOT cruises [46]. The observational data for the AS site (Supplementary Table S3) were obtained from the Joint Global Ocean Flux Study (JGOFS) (see http://usjgofs.whoi.edu/jg/dir/jgofs/, accessed 30 June 2022).

The differences between observations ($\hat{a}$) and model outputs (a) were evaluated using a cost function (J):

(1) $$J=\overset{M}{\underset{m=1}{\sum }}\frac{1}{N_{m}}\overset{N_{m}}{\underset{n=1}{\sum }}{\left(\frac{a_{m,n}-{\hat{a}}_{m,n}}{\sigma_{m}}\right)}^{2}$$

where m and n represented the observational data types and data points, respectively, M was the total number of the observational data types, $N_{m}$ was the total number of the observational data points of data type m, and $\sigma_{m}$ was the target error for data type m, which, in general, was the standard error of the observations for each data type. That is, a model output $a_{m,n}$ was considered to perfectly fit its corresponding observational datum ${\hat{a}}_{m,n}$ when their difference was no more than $\sigma_{m}$, although this could not be realized in most cases. A lower cost function indicated a better match of the model to the observations. An ideal model fit to a type of observation could have the corresponding component of the cost function as less than 1. Initially, all the parameters were optimized from the initial guesses of values based on historical data. Those parameters with unrealistic optimized values were reset to their initial values and removed from the optimization, while the realistic optimized values were kept. This process was carried out iteratively until all the optimized parameter values were realistic. The parameters with acceptable levels of uncertainty were marked as “optimized”, while those with high uncertainty (but with realistic optimized values) were marked as “adjusted”. More details of the optimization are described elsewhere [43].

### 2.3. Viral Module

The viral module constructed in the present study and incorporated into the standard model included three processes (Figure 1b): (1) the mortality of heterotrophic bacteria caused by viral lysis, which contributed to the production of viruses and LDOC and to an implicit loss term to RDOC; (2) viruses released from lysed heterotrophic bacteria; (3) the decay of viruses, which was assumed to contribute to the LDOC pool. Hence, the module only represented the viral infection of heterotrophic bacteria but not autotrophic phytoplankton, considering that the viral-induced mortality of heterotrophic bacteria (simply termed “bacteria” hereafter) was the highest compared to other organisms in the ocean [2,10]. The temporal change rate of the bacterial carbon biomass ($C_{BA}$) was calculated as follows:

(2) $$\frac{{\mathrm{dC}}_{BA}}{\mathrm{dt}}=GROW_{BA}^{C}-RESP_{BA}^{C}-EXCR_{BA}^{SDOC}-GRAZ_{BA}^{C}-MORT_{BA}^{C}$$

where the terms on the right-hand side were the gross growth (uptake of organic matter), respiration, excretion of SDOC, grazing by protozoan zooplankton and mortality caused by viral lysis of bacteria. The carbon-based mortality term $MORT_{BA}^{C}$ was calculated by first parameterizing the mortality in the unit of bacterial abundance:

(3) $$MORT_{BA}^{A}=r_{INFE,BA}\;\cdot \;A_{VA}\;\cdot \;A_{BA},$$

in which $A_{VA}$ is viral abundance, $A_{BA}$ is bacterial abundance converted from the C biomass using a commonly used conversion factor ($q_{BA}$) of 10 fg C cell^−1^ [47] and $r_{INFE,BA}$ is the viral infection rate on bacteria. As the viruses were explicitly and dynamically simulated, the VMM rate (here equaling $r_{INFE,BA}\;\cdot \;A_{VA}$) was then proportional to viral abundance and varied in space and time in our viral module. $MORT_{BA}^{A}$ was then converted back to the unit of carbon biomass:

(4) $$MORT_{BA}^{C}=q_{BA}\;\cdot \;MORT_{BA}^{A}$$

In this scheme, the virus-mediated mortality rate of bacteria, i.e., the mortality divided by biomass, therefore, was not constant but was proportional to viral abundance. Note that although the parameter $r_{INFE,BA}$ was not optimizable in our data assimilation procedure, it was tuned to ensure that the bacterial mortality rates in the model with the viral module, after being averaged over the model domain, were close to the constant mortality rate used in the standard model. More about this setup will be discussed later.

When bacteria were lysed, viruses were released at a certain burst size ($bs_{BA}$), which was the number of viruses released by each lysed bacterium:

(5) $$GROW_{VA}^{A}=bs_{BA}\;\cdot \;MORT_{BA}^{A}$$

A burst size ($bs_{BA}$) of 23 was initially used at the AS site based on the mean value measured in the nearby estuarine sites [32,48]. This value was close to the average burst size of 24 in marine environments [49]. A smaller burse size of 15 was initially set at the HOT site by considering that it is more oligotrophic than AS and viral replication in infected bacteria can be limited by nutrients in oligotrophic open oceans [50]. Additionally, the burst size of 15 used at HOT was within the range (12 to 20) found in the coastal North Pacific [6,51]. It was slightly adjusted to 15.7 by the data assimilation at HOT, but could not be adjusted by the data assimilation at AS.

The viruses decayed using the following scheme:

(6) $$DECAY_{VA}^{A}=d_{VA}\;\cdot \;A_{V}{}^{2},$$

where $d_{VA}$ is a parameter for the decay. This scheme, which was also used in a previous marine ecological model [25], introduced a density-dependent decay rate for viruses, with the viral decay rate equaling $d_{VA}\;\cdot \;A_{V}$ and, therefore, increasing with viral abundance. We used this scheme to implicitly represent how marine viruses were also removed by grazing [52] or by aggregating into large particles [53], while the density of grazers and large particles increased with viruses. The values of $d_{VA}$ (Table 2) were unknown and were tuned in this study for reasonable viral abundance. The realized decay rates using the selected values of $d_{VA}$ are evaluated in the Discussion. It is worth noting that non-density-dependent viral decay, i.e., using a constant viral decay rate, is, however, a more commonly used scheme in previous marine ecological models [40]. How the different schemes can impact the model dynamics may need further investigation.

The time rate of viral biomass ($C_{VA}$) was then:

(7) $$\frac{dC_{VA}}{\mathrm{dt}}=q_{VA}\;\cdot \;\left(GROW_{VA}^{A}-DEVAY_{VA}^{A}\right),$$

where $q_{VA}$ is the carbon content per single virus of 0.05 fg C per viral particle based on a previous empirical relationship between the carbon content ($C_{head}$, carbon atoms) and radius (r, nm) in the viral head [12]:

(8) $$C_{head}=41{\left(r-2.5\right)}^{3}+130\left(7.5r^{2}-18.75r+15.63\right)$$

By using 20–40 nm for the viral radius, a range of most viruses found in the Tara Ocean Expedition [54], each viral particle content was 0.01–0.07 fg C.

In addition to the production of viruses, a small fraction of carbon from bacterial mortality ($MORT_{BA}^{C}$) also contributed to RDOC, and the rest contributed to LDOC.

The parameters used in the viral module are listed in Table 2. The full list of model parameters, including their initial values and their optimized or adjusted values, if applicable, can be found in Supplementary Table S1.

In this study, uncertainties of 1 standard deviation were reported with corresponding averages, unless mentioned otherwise.

To prevent visual distortion of the data and exclusion of readers with color vision deficiency, scientific color maps were used in several figures [55].

## 3. Results

### 3.1. Model Performance

The incorporation of our viral module into the standard model slightly increased the model fit to the assimilated observations, resulting in lowered cost functions (Equation (3)), i.e., the model fitting better to observations, for more than half of the types of observations at both HOT and AS (Table 1). An exception was at AS, where the model with the viral module (termed “viral model” for simplification hereafter) had a slightly worse fit to the biomass of bacteria (Table 1). Overall, the changes in the cost function were relatively small, revealing that the viral module only slightly improved the model performance in terms of fitting observations. This was not surprising because, as described above, we tuned the model so that the incorporation of the viral module did not substantially change the overall level of bacterial mortality.

### 3.2. Average Model Results

**Figure 2:**
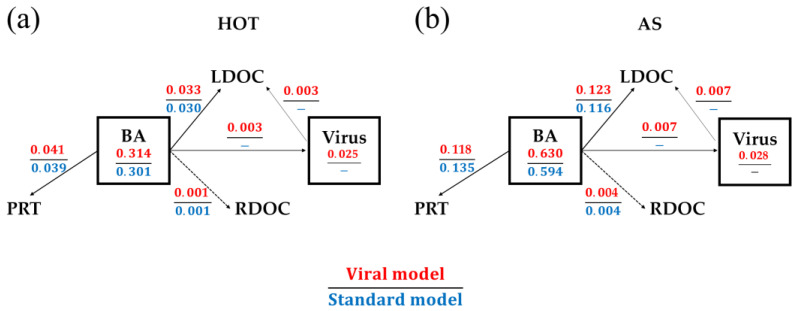
Average model results related to viral processes. (**a**) The results for the HOT site, and (**b**) the results for the Arabian Sea site. Each pair of numbers shows the results of the model with the viral module (upper red numbers) and the standard model without it (lower blue numbers). The values in boxes are standing stocks, and those on arrows are flows. BA: heterotrophic bacteria; LDOC and RDOC: labile and refractory DOC; PRT: protozoan zooplankton. Units: mmol C m^−3^ for standing stocks and mmol C m^−3^·d^−1^ for flows.

**Figure A1:**
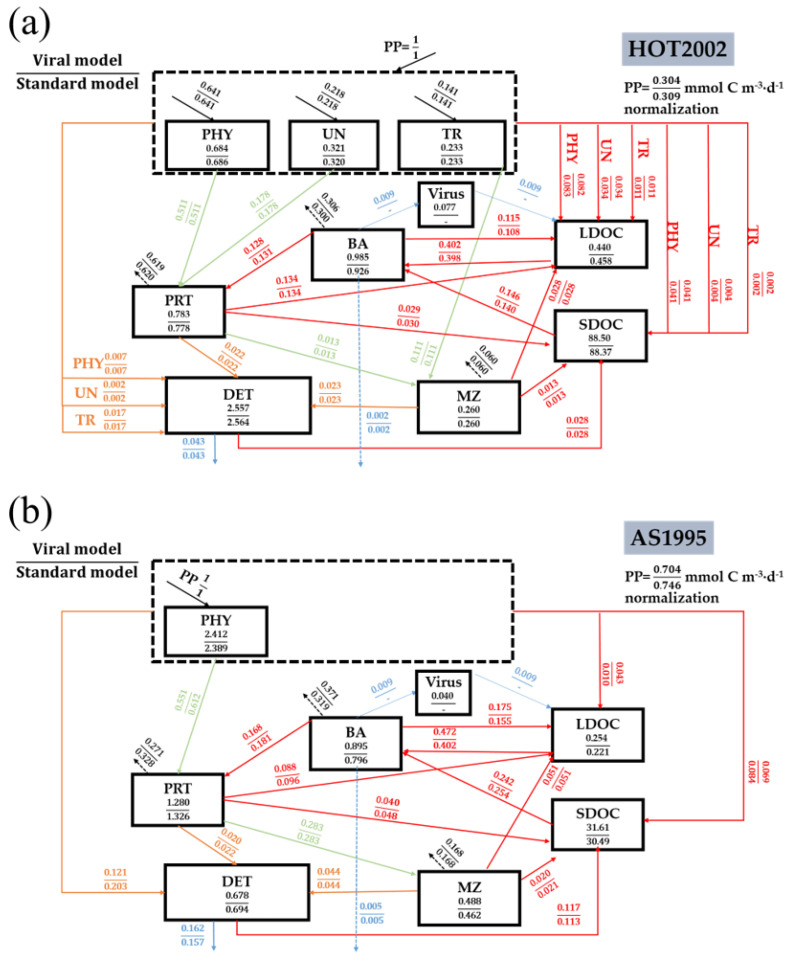
Modeled average carbon fluxes (arrows) and stocks (boxes), normalized to primary production (stocks normalized to the amount of primary production in one day) at (**a**) the HOT site in 2002 and (**b**) the Arabian Sea site in 1995.

The modeled standing stocks and flows averaged over the model’s entire temporospatial domain can be found in Figure A1. Here, we focus on the average model results related to the processes of viruses and their hosts, bacteria (Figure 2). The model showed that 11.8% of bacteria per day were lysed by viruses at HOT, while a comparable fraction (13.1% d^−1^) was grazed by protozoan zooplankton (Figure 2a), which was consistent with previous studies suggesting that viruses and grazing contribute about equally to the loss of bacteria [38,56]. At AS, the productivity was higher and organisms were more active (Figure A1), which led to higher removal rates of bacteria than those at HOT, including 21.3% d^−1^ lysed by viruses and 18.7% d^−1^ grazed by protozoan zooplankton (Figure 2b). At both sites, approximately 90% of the lysed biomass of bacteria was transformed to labile DOC, which, therefore, can be quickly recycled, while the rest became viral biomass or refractory DOC (Figure 2). The production of viruses was balanced by their decay over the model domain.

In the standard model in which a constant mortality rate was applied to bacteria to implicitly represent viral lysis, lysis and grazing removed 10.3% d^−1^ and 13.0% d^−1^ of bacterial biomass at HOT and 20.2% d^−1^ and 22.7% d^−1^ at AS, respectively (Figure 2). These fractions were at the same levels as those obtained in the viral model at each site, which, as described in Materials and Methods, was realized by tuning the model parameters.

### 3.3. Spatiotemporally Variable Simulation of Viruses and Their Induced Bacterial Mortality

**Figure 3:**
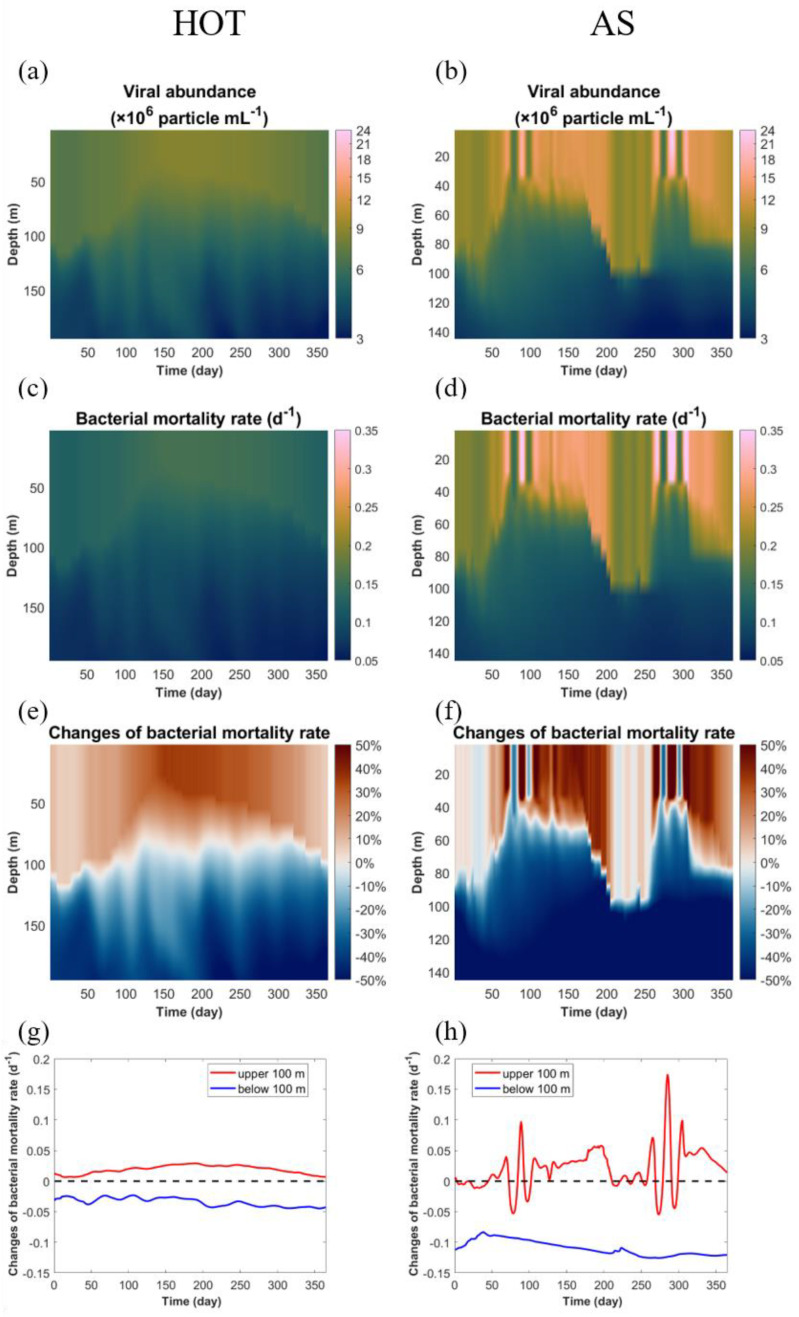
Results of modeled viral abundance and its mediated bacterial mortality. The modeled viral abundance (**a**,**b**), the virus-mediated mortality (VMM) rates of heterotrophic bacteria (**c**,**d**), the relative changes of the VMM rates in the viral model compared to those in the standard model over the model domain (**e**,**f**) and in surface (red lines) (upper 100 m) and subsurface (blue lines) (below 100 m) layers (**g**,**h**) are shown for the HOT (**a**,**c**,**e**,**g**) and Arabian Sea (**b**,**d**,**f**,**h**) sites. Note that in (**f**), the range of the color bar is limited to ±50% for clearer comparison, although some data are out of this range.

At HOT, the modeled viral abundance varied from 3 × 10^6^–9 × 10^6^ mL^−1^ (Figure 3a), which was within the range of the observed viral abundance in the open ocean [1] and was also comparable to previous measurements at HOT [57]. The viral abundance in the surface layer (defined as the upper 100 m in the present study) at HOT was several times higher than that in the subsurface layer (defined as below 100 m in the present study) (Figure 3a), which was consistent with previous analyses of the vertical distribution of viruses in the North Pacific [1], reflecting decreasing productivity from the surface to the deep ocean. The modeled viral abundance at the surface was the highest in summer, while the depth that high viral abundance reached was also shallower in summer because of stronger stratification (Figure 3a). There was no clear seasonal variability in viral abundance in the subsurface layer (Figure 3a).

At AS, the modeled viral abundance in the surface layer (Figure 3b) was substantially higher (5 × 10^6^–24 × 10^6^ mL^−1^) than at HOT and had stronger temporal variations, particularly several sequential short-term events with quickly elevated and reduced viral abundance during days 80–100 and days 260–300. In the subsurface layer, the viral abundance at AS was comparable to, sometimes even lower than, that at HOT (Figure 3a,b). In contrast to HOT, there were two seasons with relatively high viral abundance at AS in that year, including that from spring to early summer and that in early winter, probably because of the seasonable monsoons that occurred in this region.

The bacterial mortality rates caused by infection of viruses, i.e., the VMM rates, in the viral models showed substantial dynamics both in space and time, ranging mostly in 0.05–0.15 d^−1^ and 0.05–0.30 d^−1^ at HOT and AS, respectively (Figure 3c,d). In our viral module, the VMM rate, as described above, was proportional to the viral abundance in our viral module. In comparison, in our standard model, as well as in most other marine ecological models, the VMM rates were set as constant by model parameters.

At HOT, the viral module increased the VMM rates relative to 0.103 d^−1^ used in the standard model by up to 25% on average in the surface layer, but decreased them by approximately 20–40% in the subsurface layer (Figure 3e,g). This was understandable because the viral abundance was higher at the surface than in the subsurface (Figure 3a). The VMM rates in the surface layer showed a clear seasonal cycle, high in summer and low in winter, but no clear patterns in the subsurface layer (Figure 3g), which was also consistent with the pattern of modeled viral abundance.

At AS, the viral model produced a more dynamic field of VMM rates than at HOT (Figure 3d), which could be up to 150% higher or 70% lower than the constant rate (0.202 d^−1^) used in the standard model (Figure 3f). Temporally, the viral module could greatly change the average VMM rates in the surface layer, from −35% to up to +80%, compared to the constant rate in the standard model (Figure 3g). For comparison, in the subsurface layer, the VMM rates in the viral module were relatively stable over time, at ~50–60% lower than the constant rate of the standard model (Figure 3g).

### 3.4. Cascading Effects of the Viral Module

The viral module can have cascading effects through modeled trophic levels. The most direct impact of the dynamic VMM rates was apparently on the modeled bacterial abundance. Bacteria are an important player in recycling nutrients in marine ecosystems and they were too in our model [43,58]. When the implementation of the viral module caused dynamics in bacterial mortality and abundance, it also changed the recycling efficiency of nutrients. Consequently, our modeled primary production changed with the modified rates of nutrient recycling by the viral module, which, in turn, could impact the carbon export to the deeper ocean.

**Figure 4:**
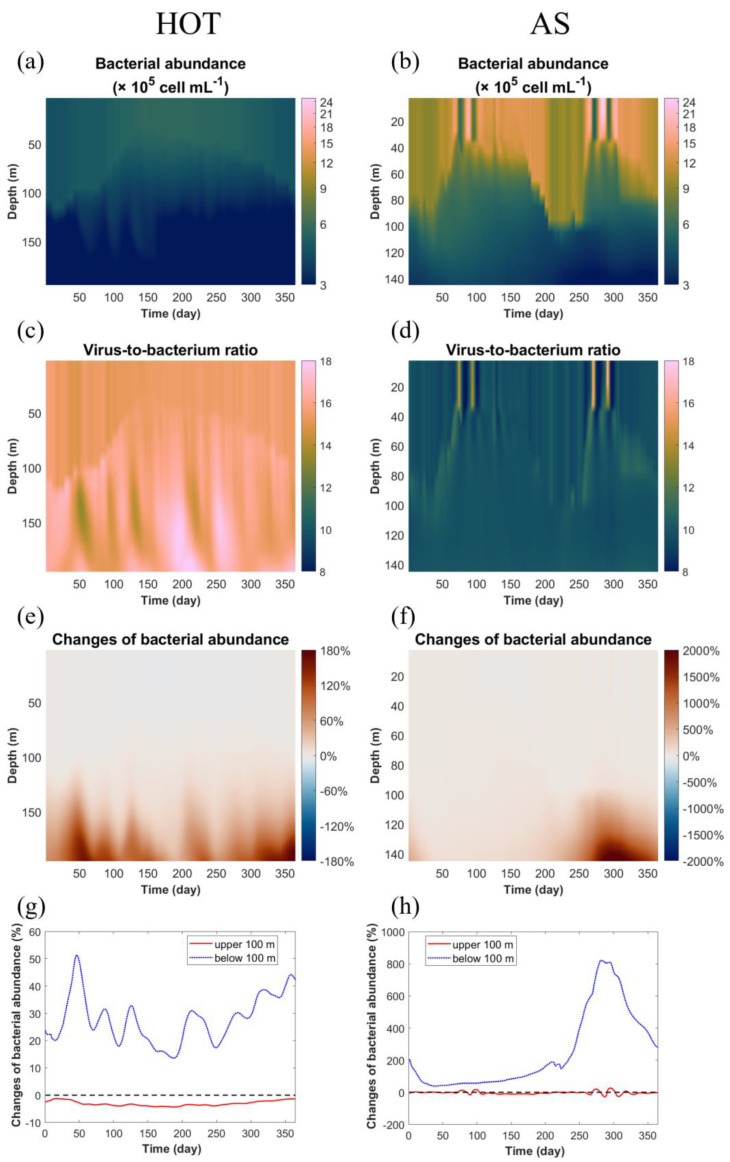
Modeled heterotrophic bacterial abundance. The modeled heterotrophic bacterial abundance (**a**,**b**) and the modeled virus-to-bacteria ratio (**c**,**d**) are shown. The relative changes in modeled bacterial abundance in the viral model compared to those in the standard model are also shown over the model domain (**e**,**f**) and in surface (red lines) (upper 100 m) and subsurface (blue lines) (below 100 m) layers (**g**,**h**). (**a**,**c**,**e**,**g**) HOT site; (**b**,**d**,**f**,**h**) Arabian Sea site. Note the different scales between (**e**) and (**f**) and between (**g**) and (**h**).

First, we analyzed the modeled bacterial abundance (Figure 4a,b). This generally had a similar pattern to the modeled viral abundance at both sites, indicating close interactions between bacteria and viruses through the production of viruses from bacteria and the lysis of bacteria by viruses. However, the virus-to-bacterium ratio (VBR) showed some variations. Overall, the VBR at HOT was higher than that at AS (Figure 4c,d). At HOT, the VBR increased with depth (Figure 4c), which was consistent with typical observations in oligotrophic oceans [59,60]. In comparison, the VBR at AS generally lacked vertical dynamics, except during those short-term events described above when VBR, viral abundance and bacterial abundance all changed quickly but in opposite directions (Figure 4d). These short-term events clearly showed that VBR was reduced when both bacterial and viral abundance increased, and vice versa, which was consistent with the pattern found in a meta-analysis of historical data [61].

**Figure 5:**
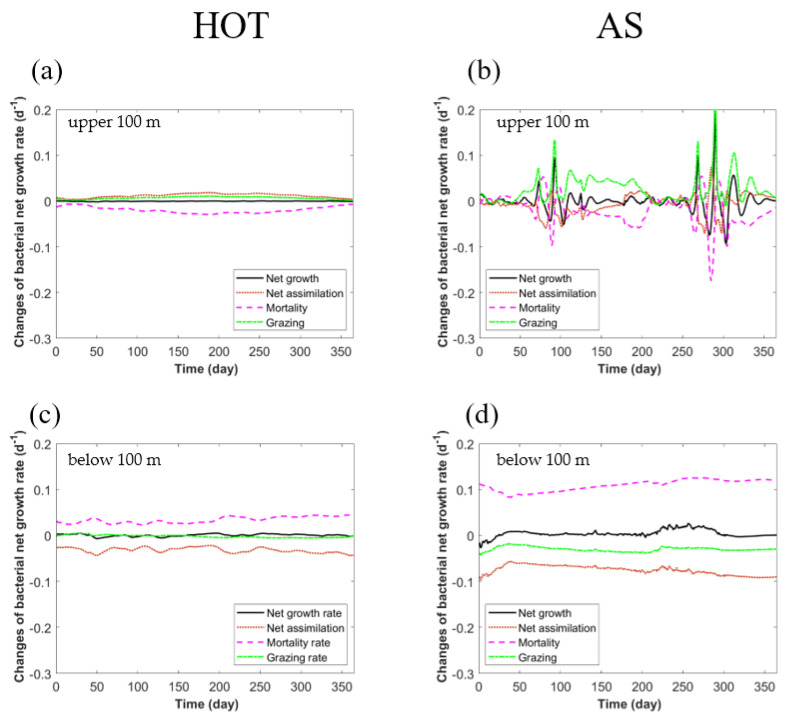
The impact of the viral module on the modeled heterotrophic bacterial growth rate. The results are the changes in the heterotrophic bacterial net growth rate attributed to net assimilation (gross growth minus respiration), mortality and zooplankton grazing in the surface (upper 100 m) (**a**,**b**) and subsurface (below 100 m) layers (**c**,**d**). (**a**,**c**) HOT site; (**b**,**d**) Arabian Sea site.

We then analyzed the changes in bacterial abundance caused by the viral module. At HOT, compared to the standard model, the viral module changed the bacterial abundance by -5% to +190% (Figure 4e). In the surface layer, the viral module only reduced the average bacterial abundance by 3.1% ± 0.9% (Figure 4g), which was much weaker than the 16% ± 6% increase in the bacterial mortality rate (Figure 3g). This was because the increased VMM rate (by 0.019 ± 0.007 d^−1^) was largely compensated for by both the elevated net assimilation (gross growth minus respiration) rate of bacteria (by 0.012 ± 0.004 d^−1^) and the decreased grazing rate on bacteria (by 0.007 ± 0.003 d^−1^), resulting in a much weaker change in the net growth rate of bacteria (Figure 5a). The elevated bacterial growth rate in the viral model was mainly because the higher production of labile dissolved organic matter (DOM) from the bacteria, due to their higher mortality rate, in turn, supported the growth of bacteria themselves (Figure 2a). In other words, the viral module caused a faster recycling of organic matter in the microbial loop in the surface layer, which could partly compensate for the higher VMM rates of bacteria. Meanwhile, the decreased grazing rate on bacteria resulted from the reduced bacterial abundance based on the scheme used in our model, in which the grazing rate was dependent on the densities of prey and predator [43]. Similarly, in the subsurface layer, the benefit that the bacteria obtained from the reduced VMM mortality rate (by 0.034 ± 0.007 d^−1^) was largely offset by the reduced bacterial net assimilation (by 0.032 ± 0.006 d^−1^), while the changes in grazing (0.002 ± 0.003 d^−1^) were much weaker than other factors because of the already low grazing rate in this layer (Figure 5c).

At AS, the viral module changed the bacterial abundance in the range of −40% to +2000%, i.e., more greatly than at HOT (Figure 4e,f), which was expected considering that the seasonal monsoons in the Arabian Sea generated stronger variability in environmental conditions and modeled VMM rates (Figure 3). Similar to HOT, the changes in VMM rates at AS could be partly offset by the changes in the bacterial net assimilation rate and the grazing rate on bacteria, while the temporal variability was greater at AS than at HOT, resulting in much fewer changes in growth rates (Figure 5b,d). This suggested that the internal interactions of different players in the ecosystem could largely stabilize the dynamic VMM rates introduced by the viral module. However, even a small change in the net growth rate could accumulate and result in much larger changes in bacterial abundance (Figure 4e–h).

**Figure 6:**
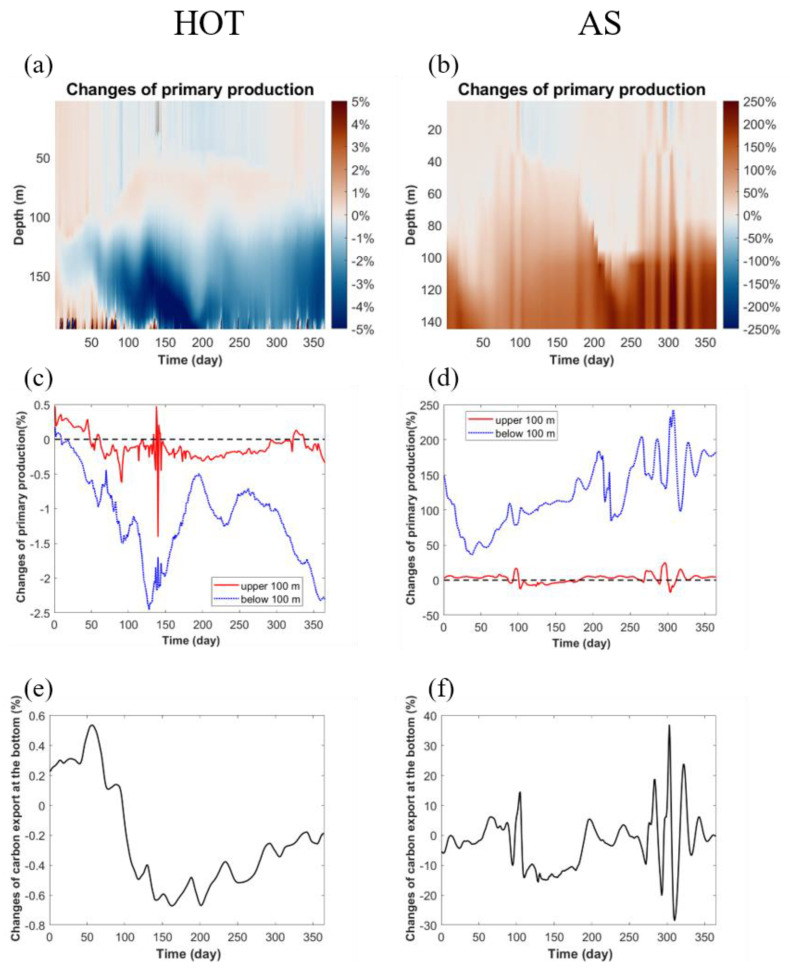
The impact of the viral module on the modeled primary production and carbon export. The relative changes in primary production in the model with the viral module compared to those in the standard model are shown (**a**,**b**) over the full model domain and (**c**,**d**) the averages in surface (red lines) (upper 100 m) and subsurface (blue lines) (below 100 m) layers. The relative changes in carbon export at the bottom of the model domain (**e**,**f**) are also shown. (**a**,**c**,**e**) HOT site; (**b**,**d**,**f**) Arabian Sea site. Note the different scales between the two sites.

The relative changes in primary production at HOT were very small (<±5%), while those at AS were much stronger (up to 200%) (Figure 6a–d). This was because the recycling of nutrients by bacteria was more active at AS due to its much higher bacterial production than at HOT [43] (Supplementary Tables S2 and S3). Another reason was that, as discussed above, the viral module also impacted AS more strongly than HOT on bacterial mortality and abundance (Figure 3 and Figure 4). The higher relative changes in primary production in the subsurface than in the surface layers at AS (Figure 6b,d) were also consistent with the vertical variation in the viral impacts on bacterial mortality and abundance (Figure 3f and Figure 4f). Interestingly, the decreased bacterial mortality in the subsurface layer when implementing the viral module (Figure 3e,f) can differently lead to decreased and increased primary production at HOT and AS, respectively (Figure 6a–d). The directly released labile DOM from dead bacteria can be remineralized by other bacteria to nutrients, contributing positively to primary production. Meanwhile, the reduced bacterial abundance may slow the remineralization efficiency of organic matter produced by other organisms in the ecosystems. Our model results then tentatively revealed complicated cascading consequences in ecosystems with different biogeochemical characteristics. As a consequence of the changed primary production, the viral module did not substantially impact the carbon export at the bottom of the model domain at HOT (<±1%), while at AS, the module changed the carbon export by up to nearly 40% (Figure 6e,f).

## 4. Discussion

In this study, we constructed a module with explicit representations of marine viral processes and combined the module with a marine ecological model. The model considered the infection of marine viruses on their major hosts, bacteria, assuming that the VMM of bacteria and the production of viruses were determined by the contact rates between viruses and bacteria. In addition to an explicit simulation of viral abundance, the most significant difference between our constructed viral module and other marine ecological models was the spatiotemporally variable VMM. The model was then applied to two open-ocean sites with contrasting biogeochemical and ecological properties in the North Pacific Subtropical Gyre and the Arabian Sea using a one-dimensional vertical framework covering the euphotic zone. Previous ecological models involving marine viruses were either theoretical studies not simulating specific ocean regions driven by realistic dynamic conditions [40,41,42], or did not simulate spatiotemporally variable VMM rates of microorganisms [25]. The present study, to our knowledge, was the first effort to implement spatiotemporally variable VMM rates in marine ecological models driven by realistic dynamic conditions.

The performance of the viral module was assessed by comparing the results of the model cases with and without the viral module. To achieve a more objective and reliable assessment, two efforts were made. First, the two model cases were optimized to best fit the observations using a data-assimilation approach, resulting in different sets of parameter values that were adjusted objectively in each model case. In such a way, biases that can be introduced by using the same set of parameter values under different model structures, if data assimilation is not conducted, can be largely avoided. Second, because the viral module generated spatiotemporally variable VMM rates of bacteria, the model parameter for viral infection was tuned to ensure that the average VMM rate was close to the constant rate used in the standard model. Therefore, with overall similar VMM levels in the two model cases, systematic differences caused by VMM can be largely avoided and our assessment can then focus on spatiotemporally variable anomalies in model results generated by the viral module. Although the implementation of the viral module only slightly increased the model performance in fitting the discrete observations, one of the main benefits of the viral module was to produce high-frequency dynamic results, particularly in highly productive ocean regions. High-frequency sampling could be a useful way to better evaluate the performance of explicit representations of marine viruses.

Since the typical VBR is assumed to be 10 [7,61], we can replace the density of viruses with 10 times the density of bacteria (i.e., $A_{VA}=10A_{BA}$) at all times. In this way, we can bypass Equation (7) and plug that term directly in Equation (3) to generate a density-dependent term that mimics the “kill-the-winner” mechanism. This is a method that can be easily implemented in marine biogeochemical models without considerably modifying the model, providing the potential to improve the model performance on bacteria. The approach, for example, has been used in global-scale marine ecological modeling [62]. However, a meta-analysis of historical data showed a wide range of VBR in the ocean and suggested that VBR generally decreases with increasing bacterial abundance [61]. Our modeled VBR was consistent with this pattern, showing the lower VBR at AS, where productivity was higher, than at the more oligotrophic HOT, as well as the increasing VBR with depth, particularly at HOT (Figure 4c,d). In other words, compared to just using a bacterial density-dependent term for VMM in which constant VBR is implicitly assumed, an explicit representation of viral processes in models driven by dynamic conditions, such as those created in this study, decouples viral abundance from bacterial abundance, and therefore, generates spatiotemporally variable VBR. This is another reason why the explicit representation of viral processes in marine ecological models could be used to further improve the model performance on VBR, and therefore, on bacterial mortality in realistic dynamic conditions.

In our viral module, the contact rate between viruses and their major hosts, heterotrophic bacteria, determined the infection and subsequent mortality of the hosts. The model generated a dynamic mortality rate of heterotrophic bacteria over space and time, increasing the mortality when the bacteria were more abundant, such as in surface layers and highly productive seasons/sites, while reducing the mortality in subsurface layers or when productivity was low. Therefore, when bacterial abundance in our model increased with elevated supplies of organic matter under higher productivity, more viruses were produced, and the bacterial mortality rate also increased. Our viral module thus tended to suppress the variability of bacterial abundance to some degree (Figure 4a,b), a mechanism consistent with the “kill-the-winner” hypothesis on the roles of marine viruses [11]. In comparison, a model using a constant bacterial mortality rate could not test this hypothesis. The explicit representation of viral processes tends to weaken the spatiotemporal variability of heterotrophic bacteria in the model. Although the variations in heterotrophic bacterial mortality rates in the model can be partly compensated for by the changes in bacterial net assimilation and their removal by zooplankton, they still cause substantial changes in heterotrophic bacterial abundance, primary production and carbon export.

The removal of viruses by their decay can also contribute to the dynamics of viral abundance, potentially decoupling the bacterial and viral abundances. Using the current scheme and parameters of our viral module, the model simulated viral decay rates of 13.7% ± 0.7% d^−1^ and 8.3% ± 0.7% d^−1^ in the surface and subsurface layers at HOT, respectively, while the rates were 40.6% ± 7.8% d^−1^ and 20.1% ± 2.9% d^−1^ at AS. These decay rates at AS were generally comparable to the observations of 39.4% ± 5.0% d^−1^ measured in the western Pacific Ocean [63], 43.9% ± 10.2% d^−1^ in the Central Adriatic Sea [34], 25.4% ± 8.4% d^−1^ in the North Sea [64] and 70.8% ± 30.1% d^−1^ in the South China Sea [31]. The low viral decay rates in extremely oligotrophic open oceans such as HOT can be expected because of their low productivity, but there have been no measurements reported in this kind of ocean environment.

Our viral scheme was an initial step in implementing explicit and dynamic simulations of viruses and their interactions with their hosts in the ocean. The scheme only considered the contact rate between viruses and bacteria and assumed that the infection rate by viruses was proportional to the contact rate. The theoretical contact rate between spherical viruses and bacteria is [41,65]:

(9) $$C_{s}=2\pi \cdot d_{BA}\cdot D_{VA}\cdot A_{VA}\cdot A_{BA},$$

where $d_{BA}$ is the bacterial diameter and $D_{VA}$ is the viral diffusivity. Only some of the contact events between viruses and bacteria can seemingly result in successful infections. Therefore, a comparison between Equations (3) and (9) indicates that the infection parameter $r_{INFE,BA}$ must be lower than a contact-based upper bound of $2\pi \cdot d_{BA}\cdot D_{VA}$, which, by using a $d_{BA}$ of 0.6–1.0 μm and $D_{VA}$ of 3–15 μm^2^ s^−1^ [65], ranges from 1 × 10^−12^–8 × 10^−12^ m^3^ d^−1^. By fitting average VMM rates, as noted above, the values of $r_{INFE,BA}$ used in our model at HOT and AS (1.82 × 10^−14^ m^3^ d^−1^ and 2.17 × 10^−14^ m^3^ d^−1^, respectively; Table 2) were two orders of magnitude lower than the contact-based upper bound. In other words, our model estimates that after a virus contacts a bacterium, the possibility of a successful infection is in the order of 1%.

However, this possibility can vary substantially as a successful viral infection also depends on other factors. Ultraviolet radiation can decrease viral infectivity by degrading viral proteins [66]. The frequency of infected host cells was negatively correlated with salinity along the coast of Senegal, where the range of salinities (10–360) was wide [67]. Additionally, our model does not represent the more recently proposed “piggyback-the-winner” model, in which temperate viruses can switch from lytic infection to lysogenic infection, and therefore, coexist and replicate with their hosts, particularly when the host bacteria become more abundant [68,69]. Our simplified contact-rate-based model then may overestimate the VMM rates, particularly at a high bacterial abundance.

Furthermore, the host physiology can also impact the production rate of viral particles by changing the latent time and the burst size of viruses. A larger burst size was generally associated with a longer latent time [70], and the viral burst size was found to depend on the bacterial growth rate [71]. The length of latent time was also found to be positively correlated with the degree of P limitation [72]. The importance of the host physiology for viral processes has further been revealed by several modeling studies [73,74].

The decay of viruses in the ocean can also substantially impact their abundance [75] and consequently the rate of infection on hosts. The decay rate of viruses in our module only depended on viral abundance, but it can also be greatly elevated by ultraviolet light radiation [63,66,76,77,78], by attaching to particles [36,76], and by increasing temperature [63,76].

Last, our model only represented the viral infection of heterotrophic bacteria because of their relatively high virus-mediated mortality compared to that of phytoplankton in the ocean [2,10]. Yet, viral infection can also be important to impact the abundance and biogeography of phytoplankton [79,80,81].

The representation of the above processes and factors in models will need considerable work in the future, and their importance for model performance could also be evaluated. Nevertheless, even by using this simplified scheme to represent processes involving marine viruses, our study demonstrated that it already, depending on environmental conditions, generates substantial model dynamics, not only in the hosts but also in other functions of the ecosystems, including primary production and carbon export.

## 5. Conclusions

This study was the first effort to explicitly simulate marine viruses and their related processes in marine ecological models driven by realistic biogeochemical conditions that varied in space and time. The explicit representation of viruses and their related processes generated substantial dynamics in model variables when the model was run in dynamic marine conditions. One of the most significant impacts of the viral module was the decreasing VMM rates with depth, leading to a higher bacterial abundance in the deeper waters in the model. Thus, the model with explicit viral processes would allow a stronger remineralization of organic matter in the subsurface. Our modeled results also showed that the viral module, by regulating the nutrient remineralizer, bacteria, can have cascading effects on other components of the simulated ecosystems, such as the primary production and the carbon export. Overall, our study suggests that the explicit representation of viral processes in ecological models, in particular, the dynamic simulation of virus-mediated mortality, is more important in high-productivity oceans where nutrient recycling by bacteria is more active, and in subsurface layers where virus-to-bacterium ratios are high. It is worth including more viral processes and hosts in future modeling studies to improve the model performance and better assess the roles of viruses in marine ecosystems.

## Figures and Tables

**Table 1:** Comparison of the cost functions between the standard model and the viral model at the HOT and AS sites. Cost functions substantially different between the two models are marked with bold fonts. MZc: C biomass of metazoan zooplankton; PHYn: phytoplankton N biomass; CHL: chlorophyll a; PP: primary production; BAc: C biomass of heterotrophic bacteria; BP: heterotrophic bacterial production: sDOC, sDON, sDOP: semilabile dissolved carbon, nitrogen, phosphorus; POC, PON, POP: particulate organic carbon, nitrogen, phosphorus; STc, STn, STp: C, N, P flux collected by sediment traps.

	NO_3_^−^	PO_4_^3−^	MZc	PHYn	CHL	PP	BAc	BP	sDOC
HOT site									
Standard model	**1.2**	0.4	**1.2**	1.4	**9.1**	24	11	**2.3**	**1.8**
Viral model	**0.9**	0.4	**1.1**	1.4	**8.9**	24	11	**1.7**	**1.5**
AS site									
Standard model	**6.8**	**1.7**	**1.4**	38	4.4	10.6	**5.5**	**7.3**	**3.1**
Viral model	**5.9**	**1.4**	**1.3**	38	4.3	10.4	**6.3**	**6.9**	**3.3**
	sDON	sDOP	POC	PON	POP	STc	STn	STp	**Total**
HOT site									
Standard model	**1.5**	**1.9**	3	1.7	**1.7**	0.8	**0.9**	0.56	**64.9**
Viral model	**1.4**	**1.8**	3	1.7	**1.6**	0.8	**0.9**	0.56	**62.7**
AS site									
Standard model	**1.7**		2.4	5.0		**5.4**	**4.7**		**97.4**
Viral model	**1.5**		2.4	5.0		**4.9**	**4.3**		**95.7**

**Table 2:** Symbols, units and descriptions of viral module parameters.

Symbol	Value at HOT	Value at AS	Unit	Description
$r_{INFE,BA}$	1.82 × 10^−14^	2.17 × 10^−14^	m^3^ particle^−1^ d^−1^	Viral infection rate on heterotrophic bacteria
$bs_{BA}$	15.7	23	unitless	Burst size
$d_{VA}$	1.84 × 10^−14^	5.11 × 10^−14^	m^3^ particle^−1^ d^−1^	Parameter for viral decay
$q_{VA}$	0.05	0.05	fg C particle^−1^	Carbon content per virus
$q_{BA}$	10	10	fg C cell^−1^	Carbon content per heterotrophic bacterial cell

## Data Availability

All the data are shown in the manuscript and can be obtained from the authors on request.

## Supplementary Materials

The following supporting information can be downloaded at: https://www.mdpi.com/article/10.3390/v14071448/s1. Table S1. Initial model parameter values before data assimilation, and optimized or adjusted model parameter values after data assimilation. Table S2. The assimilated observational data at the Hawaii Ocean Time-series Station ALOHA. Table S3. The assimilated observational data at the Arabian Sea site S7.

## Appendix A

Here, we describe the key processes of the standard model used in our study.

The standard model simulates the dynamics of carbon (C), nitrogen (N) and phosphorus (P) with variable stoichiometry in the modeled compartments (Figure 1).

Organic matter is initially synthesized to the modeled ecosystem by non-N_2_-fixing phytoplankton (PHY) and two N_2_-fixing phytoplankton, *Trichodesmium* (TR) and unicellular diazotrophs (UN). The rates of this gross growth of phytoplankton generally increase with light intensity, but can be partially inhibited when the light intensity is too high. The gross growth rates of phytoplankton groups are also controlled by their intracellular N and P quota. PHY takes up nitrate and ammonium from seawater for its intracellular N requirement and phosphate for its P requirement, the rates of which increase and saturate with the increasing concentrations of these nutrients (using a Michaelis–Menten equation). In particular, a preference for uptake is given to ammonium over nitrate. Therefore, when the nutrient concentrations are high, PHY can have a higher intracellular N or P quota and its gross growth is less limited. Meanwhile, the model sets maximal intracellular C:N and C:P ratios for phytoplankton, and gradually reduces the nutrient uptake rates when the intracellular C:N or C:P ratio increases to a value higher than the Redfield ratio (106:16 for C:N, 106:1 for C:P). The gross growth and nutrient uptake dynamics of the N_2_-fixing TR and UN groups are largely controlled by the same processes as those of PHY, except that they have a slightly higher maximal intracellular N quota and can fix N_2_ when nitrate and ammonium uptake cannot satisfy their requirements. Organic C needs to be respired to support high energy consumption by N_2_ fixation. TR and UN have lower maximal growth rates than PHY, so their advantage of fixing N_2_ in an N-depleted environment can be balanced by the advantage of PHY in that it can grow faster when N is replete. UN has different light-controlling parameter values in its gross growth, allowing it to grow in deeper, lower-lit environments than those needed by TR.

The organic matter synthesized by the phytoplankton groups is then excreted as labile and semi-labile dissolved organic matter (LDOM and SDOM), which forms sinking detritus (DET) by aggregation, respires or is grazed by zooplankton. Phytoplankton groups release 5% of their biomass per day as LDOM at the same elemental ratio as their cells, and also release 5% of their gross growth as carbohydrate (75% of LDOC and 25% of SDOC). When the phytoplankton groups have a higher intracellular C:N or C:P than the Redfield ratio, they release SDOM, which has higher C:N or C:P ratios than those in their cells. This carbohydrate and SDOM release enables the phytoplankton groups to adjust their stoichiometry to approach the Redfield ratio. Phytoplankton groups form sinking DET using a density-dependent approach, i.e., the rate of DET production increases with the density of phytoplankton. The respiration of organic carbon in phytoplankton includes a basal respiration that equals a small fraction of the biomass per day and an active respiration that is a certain fraction of the gross growth. PHY and UN are grazed by small zooplankton (PRT), while TR is grazed by large zooplankton (MZ).

In addition to PHY and UN, PRT also graze heterotrophic bacteria (BA). In addition to TR, MZ also graze PRT. Density-dependent grazing functions are used. The organic matter that PRT and MZ obtain from grazing is respired, excreted as LDOM or SDOM or forms sinking DET. The respiration of organic carbon by PRT and MZ also includes basal and active respiration. PRT and MZ release a portion of their grazed organic matter as DOM, both from sloppy feeding and from excretion, assuming 75% of DOM excreted is LDOM and the rest is SDOM. Similar to phytoplankton groups, PRT and MZ also release SDOM if C is in excess in their biomass. Additionally, they regenerate ammonium (part of which can be converted to nitrate) or phosphate if N or P is in excess, respectively, and therefore, act to remineralize organic matter. PRT and MZ also convert a fixed portion of their grazed organic matter to sinking DET at higher C:N and C:P ratios than those of their biomass, supporting our assumption that zooplankton tend to assimilate high-quality foods with lower C:N and C:P ratios. MZ is also removed by implicitly represented higher trophic levels.

A constant vertical sinking speed is assigned to sinking DET. In the model, DET dissolves and transforms to SDOM at a certain rate when it sinks.

Heterotrophic bacteria (BA) take up LDOM and SDOM. The organic matter that they obtained undergoes losses due to respiration, grazing by PRT, and mortality. The model assumes that the available labile DOC and a limited portion of SDOC are allowed for bacterial utilization. Similar to PHY, the bacterial C growth rate is limited by their cellular N and P quota. Bacteria are modeled to either take up or release ammonium and phosphate to maintain their stable elemental composition. Bacteria can take up nitrate only when other N sources are insufficient. Bacteria may also excrete SDOC if intracellular carbon is in excess. The respiration of bacterial carbon in the model also includes basal and active respiration. As described in the main text, the mortality of bacteria is at a constant rate and contributes mainly to the LDOM pool, and a small portion of loss to the implicitly represented refractory DOM pool.

The model runs in dynamic physical environments, including temperature, light and physical transfer. The rates of several key model processes increase exponentially with temperature by approximately 60% for each temperature increase of 10 °C. The temperature-dependent processes include the growth rates of phytoplankton (PHY), *Trichodesmium* spp. (TR), unicellular N_2_-fixing cyanobacteria (UN), bacteria (BA) and zooplankton (Protozoan (PRT) and Metazoan (MZ)) and basal respiration rates of BA, PRT and MZ. The intensity of light attenuates with depth when it penetrates through the water column, with the attenuation effects of water and chlorophyll considered. The materials and organisms are vertically transferred through advection and eddy diffusion. The vertical velocity used for advection is estimated from observations of the vertical isopycnal displacement. In the mixed layer, all the materials and organisms are completely mixed. Below the mixed layer depth, the diffusivity coefficient used for eddy diffusion decreases with depth, resulting in weakening eddy diffusion with depth.

The initial conditions, estimated by linearly interpolating observations over time, are set at 90 days before the simulation. The model first runs for 90 days as a spin-up period before the simulation so that the influence of the initial conditions can be largely avoided in the simulation.

At the bottom of the model, nitrate, phosphate and semi-labile DOM exchanges occur with their linearly interpolated observed concentrations immediately below the model. The exchange of other materials and organisms is ignored, assuming negligible gradients at the bottom of the model.

Further technical details of the standard model can be found in a previous study [43].
