# Supplementary material for: Assessment of Explicit Representation of Dynamic Viral Processes in Regional Marine Ecological Models
Source: Viruses. 2022 Jun 30;14(7):1448. doi: 10.3390/v14071448 (PMC9324674; doi:10.3390/v14071448)
Supplement: Supplementary file 1 [file viruses-14-01448-s001.zip › Table S1. Initial model parameter values before data assimilation and optimized or adjusted model param-eter values after data assimilation..pdf]

Table S1. Initial model parameter values before data assimilation and optimized or adjusted model parameter values after data assimilation. The confidence ranges of optimized parameters are also included. The 'Marks' indicates if the parameter was optimized with good constrains from assimilated observations (OP), or was adjusted but not well-constrained (ADJ).

Following parameters are assigned fixed values at two sites as same as Luo, *et al.* [1]

Parameter	Default value	Unit	Description
$q^{C}_{N,\min,PHY}$	0.034	mol N (mol C)$^{-1}$	Minimum N/C ratio of PHY
$q^{C}_{\mathrm{P},\min,PHY}$	0.00188	mol P (mol C)$^{-1}$	Minimum P/C ratio of PHY
$q^{C}_{\mathrm{N},\mathrm{rd}f,PHY}$	0.15	mol N (mol C)$^{-1}$	Reference (Redfield) N/C ratio of PHY
$q^{C}_{\mathrm{P},\mathrm{rd}f,PHY}$	0.0094	mol P (mol C)$^{-1}$	Reference (Redfield) P/C ratio of PHY
$q^{C}_{N,\max,PHY}$	0.17	mol N (mol C)$^{-1}$	Maximum N/C ratio of PHY
$q^{C}_{\mathrm{P},\max,PHY}$	0.0169	mol P (mol C)$^{-1}$	Maximum P/C ratio of PHY
$q^{C}_{N,\min,\mathrm{TR}}$	0.12	mol N (mol C)$^{-1}$	Minimum N/C ratio of TR
$q^{C}_{\mathrm{P},\min,\mathrm{TR}}$	0.001	mol P (mol C)$^{-1}$	Minimum P/C ratio of TR
$q^{C}_{\mathrm{N},\mathrm{rd}f,\mathrm{TR}}$	0.16	mol N (mol C)$^{-1}$	Reference (Redfield) N/C ratio of TR
$q^{C}_{\mathrm{P},\mathrm{rd}f,\mathrm{TR}}$	0.0035	mol P (mol C)$^{-1}$	Reference (Redfield) P/C ratio of TR
$q^{C}_{N,\max,\mathrm{TR}}$	0.2	mol N (mol C)$^{-1}$	Maximum N/C ratio of TR
$q^{C}_{\mathrm{P},\max,\mathrm{TR}}$	0.006	mol P (mol C)$^{-1}$	Maximum P/C ratio of TR
$q^{C}_{N,\min,\mathrm{UN}}$	0.13	mol N (mol C)$^{-1}$	Minimum N/C ratio of UN
$q^{C}_{\mathrm{P},\min,\mathrm{UN}}$	0.001	mol P (mol C)$^{-1}$	Minimum P/C ratio of UN
$q^{C}_{\mathrm{N},\mathrm{rd}f,\mathrm{UN}}$	0.16	mol N (mol C)$^{-1}$	Reference (Redfield) N/C ratio of UN
$q^{C}_{\mathrm{P},\mathrm{rd}f,\mathrm{UN}}$	0.0035	mol P (mol C)$^{-1}$	Reference (Redfield) P/C ratio of UN
$q^{C}_{N,\max,\mathrm{UN}}$	0.2	mol N (mol C)$^{-1}$	Maximum N/C ratio of UN

$q^{C}_{P,max,UN}$	0.006	mol P (mol C)$^{-1}$	Maximum P/C ratio of UN
$q^{C}_{N,BA}$	0.18	mol N (mol C)$^{-1}$	Reference N/C ratio of BA
$q^{C}_{P,BA}$	0.02	mol P (mol C)$^{-1}$	Reference P/C ratio of BA
$q^{C}_{N,PRT}$	0.2	mol N (mol C)$^{-1}$	Reference N/C ratio of PRT
$q^{C}_{P,PRT}$	0.022	mol P (mol C)$^{-1}$	Reference P/C ratio of PRT
$q^{C}_{N,MZ}$	0.2	mol N (mol C)$^{-1}$	Reference N/C ratio of MZ
$q^{C}_{P,MZ}$	0.008	mol P (mol C)$^{-1}$	Reference P/C ratio of MZ

(a) Hawaii Ocean Time-series Station ALOHA (HOT):

Following parameters were different between Standard model (or Viral model) and initial values from Luo, *et al.* [1]

.

Parameter	Initial	Standard model	Viral model	Unit	Description	Marks
μ_{PHY}	1.7097	3.1673	3.4533	d^{-1}	C-specific maximum phytoplankton (PHY) growth rate	OP
α^{CHL}_{PHY}	5.5127	6.2202	6.3335	mol C (g Chl a)$^{-1}$ d^{-1} (W m^{-2})$^{-1}$	Initial slope of Photosynthesis vs. PAR curve of PHY	OP
k^{NH4}_{PHY}	0.01	0.02	0.02	mmol m^{-3}	Ammonium half-saturation concentration for PHY growth	OP
$v^{P}_{REF,PHY}$	0.0101	0.0093	0.0106	mol P (mol C)$^{-1}$ d^{-1}	Maximum phosphorus assimilation rate per PHY carbon biomass	OP
θ	2.1289	3.6551	3.6071	g Chl a (mol N)$^{-1}$	Maximum chlorophyll a to nitrogen ratio	OP
$ex_{PSV,PHY}$	0.05	0.05	0.07	d^{-1}	PHY passive excretion rate (per biomass)	ADJ
$ex_{CHO,PHY}$	0.05	0.05	0.07		PHY carbon hydrate excretion rate (per growth rate)	ADJ

μ_{TR}	3.0	4.3366	4.3366	d^{-1}	C-specific maximum Trichodesmium (TR) growth rate	ADJ
$v_{REF,TR}^{N}$	0.4	0.4	0.3811	mol N (mol C)$^{-1}$ d^{-1}	Maximum nitrogen assimilation rate per TR carbon biomass	OP
k_{TR}^{NH4}	0.05	0.0175	0.0176	mmol m^{-3}	Ammonium half-saturation concentration for TR growth	OP
k_{TR}^{NO3}	0.5	0.0005	0.0006	mmol m^{-3}	Nitrate half-saturation concentration for TR growth	OP
pom_{TR}	0.038	0.6032	0.6032	(mmol C m^{-3})$^{-1}$ d^{-1}	POM production rate by TR	ADJ
μ_{UN}	1.6	2.6397	2.7258	d^{-1}	C-specific maximum Unicellular N2-fixers (UN) growth rate	OP
r_{SDOM}	0.00096	0.0018	0.002		parameter for SDOM's lability	OP
$resp_{\max,BA}^{A}$	0.6735	0.5491	0.5542	d^{-1}	Maximum BA growth rate	OP
$mort_{BA}$	0.049	0.1	-	d^{-1}	Bacteria mortality rate	
$r_{INFE,BA}$	1.82×10^{-14}	-	1.82×10^{-14}	m^3 particle^{-1} d^{-1}	Viral infection rate on heterotrophic bacteria	
bs_{BA}	15	-	15.72		Burst size	ADJ
d_{VA}	1.84×10^{-14}	-	1.84×10^{-14}	m^3 particle^{-1} d^{-1}	Viral decay rate	
μ_{PRT}	4.0	7.1526	7.122	d^{-1}	Protozoa (PRT) maximum growth rate	OP
g_{BA}	1.43	2.05	2.05	mmol C m^{-3}	Half-saturate density of BA in PRT grazing function	ADJ
$f_{ex,PRT}$	0.9	0.8195	0.8195		Fraction of labile DOC of total PRT DOC excretion	ADJ
$resp_{PRT}^{A}$	0.7585	0.7536	0.7485		PRT basal respiration rate	OP
μ_{MZ}	1.3	1.3	1.4958	d^{-1}	Maximum metazoa (MZ) growth rate	OP
g_{TR}	0.2007	0.1684	0.1952	mmol C m^{-3}	Half-saturate density of TR in MZ grazing function	OP
$wnsv$	5.2625	3.52	3.6972	m	Detritus sinking velocity	OP

Following parameters values are defaulted from Luo, *et al.* [1]

Parameter	Default value	Unit	Description
A_E	4000		Arrhenius parameter for temperature function
β_{PHY}	0.0005	(W m^{-2})$^{-1}$	Light inhibition parameter for photosynthesis of PHY
$v_{REF,PHY}^{N}$	0.3	mol N (mol C)$^{-1}$ d^{-1}	Maximum nitrogen assimilation rate per PHY carbon biomass
k_{PHY}^{NO3}	0.1	mmol m^{-3}	Nitrate half-saturation concentration for PHY growth
k_{PHY}^{PO4}	0.0048	mmol m^{-3}	Phosphate half-saturation concentration for PHY growth
ζ^{NO3}	2.0	mol C (mol N)$^{-1}$	Carbon requirement (respiration) to assimilate nitrate
pom_{PHY}	0.038	(mmol C m^{-3})$^{-1}$ d^{-1}	POM production rate by PHY aggregation
α_{TR}^{CHL}	0.25	mol C (g Chl a)$^{-1}$ d^{-1} (W m^{-2})$^{-1}$	Initial slope of Photosynthesis vs. PAR curve of TR
β_{TR}	0.002	(W m^{-2})$^{-1}$	Light inhibition parameter for photosynthesis of TR
$v_{REF,TR}^{P}$	0.02	mol P (mol C)$^{-1}$ d^{-1}	Maximum phosphorus assimilation rate per TR carbon biomass
k_{TR}^{PO4}	0.047	mmol m^{-3}	PO4 half-saturation concentration for TR growth
$pick_{TR}^{PO4}$	1.0	d^{-1}	Deep phosphate pick-up (recovery) rate of TR
ζ^{NFIX}	2.5	mol C (mol N)$^{-1}$	Carbon requirement (respiration) to nitrogen fixation
$ex_{PSV,TR}$	0.02	d^{-1}	TR passive excretion rate (per biomass)
$ex_{CHO,TR}$	0.05		TR carbon hydrate excretion rate (per growth rate)
$ex_{NFIX,TR}$	0.36		TR DON and ammonium release rate per nitrogen fixation
α_{UN}^{CHL}	0.5	mol C (g Chl a)$^{-1}$ d^{-1} (W m^{-2})$^{-1}$	Initial slope of Photosynthesis vs. PAR curve of UN
k_{DOM}	0.5	mmol C m^{-3}	Half-saturation concentration of available DOC for heterotrophic bacteria (BA) uptake
μ_{BA}	2.0	d^{-1}	Maximum BA growth rate

b_{RESP}	0.28	(mmol C m^{-3} d^{-1})$^{-1}$	Parameter control BA active respiration rate versus production
$ex_{ADJ,BA}$	2.0	d^{-1}	Bacteria extra semilabile DOC excretion rate
$remi_{BA}$	6.6	d^{-1}	Bacteria inorganic nutrients regeneration rate
$ex_{REFR,BA}$	0.018		Bacteria refractory DOC production rate
$f_{SLCT,BA}$	0.2		BA selection strength on SDOM
$resp_{BA}^{B}$	0.01	d^{-1}	Bacterial basal respiration rate
$resp_{\min,BA}^{A}$	0.35	d^{-1}	Bacteria minimum active respiration rate
g_{PHY}	0.7513	mmol C m^{-3}	Half-saturate density of PHY in PRT grazing function
ex_{PRT}	0.2		Total DOM excretion rate per PRT gross growth
$resp_{PRT}^{B}$	0.01	d^{-1}	PRT basal respiration rate
$ex_{ADJ,PRT}$	2.0	d^{-1}	PRT extra SDOM excretion rate
$remi_{PRT}$	4.7	d^{-1}	PRT inorganic nutrients regeneration rate
pom_{PRT}	0.027		POM production rate per PRT gross growth
g_{PRT}	1.4	mmol C m^{-3}	Half-saturate density of PRT in MZ grazing function
ex_{MZ}	0.3		Total DOM excretion rate per MZ gross growth
$f_{ex,MZ}$	0.75		Fraction of labile DOC of total MZ DOC excretion
$resp_{MZ}^{B}$	0.03	d^{-1}	MZ basal respiration
$resp_{MZ}^{A}$	0.22		MZ active respiration
$ex_{ADJ,MZ}$	2.0	d^{-1}	MZ extra semi labile DOM excretion rate
$remi_{MZ}$	4.0	d^{-1}	MZ inorganic nutrients regeneration rate
pom_{MZ}	0.15		POM production rate per MZ gross growth

$remv_{MZ}$	0.81	$(mmol\ C\ m^{-3})^{-1}\ d^{-1}$	MZ removal rate by higher-trophic levels
$f_{SDOM,HZ}$	0.1		Fraction of SDOM production by higher-trophic level ingestion
$f_{POM,HZ}$	0.14		Fraction of POM production by higher-trophic level ingestion
$q^{C}_{N,REFR}$	0.05	mol N $(mol\ C)^{-1}$	Refractory DOM N/C ratio
$q^{C}_{P,REFR}$	0.0007	mol P $(mol\ C)^{-1}$	Refractory DOM P/C ratio
$q^{C}_{N,POM}$	0.12	mol N $(mol\ C)^{-1}$	N/C ratio for POM generation by PRT and MZ
$q^{C}_{P,POM}$	0.0045	mol P $(mol\ C)^{-1}$	P/C ratio for POM generation by PRT and MZ
r_{ntrf}	0.1	d^{-1}	Nitrification rate (NH4 to NO3)
prf_{N}	1.1		Preference parameter for dissolving N content in POM
prf_{P}	4.0		Preference parameter for dissolving P content in POM
diss	0.011	d^{-1}	POM dissolution rate

(b)ARABIAN SEA (AS)

Following parameters were different between Standard model (or Viral model) and initial values from Luo, *et al.* [1]

Parameter	Initial	Standard model	Viral model	Unit	Description	Marks
μ_{PHY}	7.1098	7.1630	7.1630	d^{-1}	C-specific maximum phytoplankton (PHY) growth rate	OP
α^{CHL}_{PHY}	0.4545	0.3908	0.3908	mol C (g Chl a)$^{-1}$ d^{-1} $(W\ m^{-2})^{-1}$	Initial slope of Photosynthesis vs. PAR curve of PHY	OP
θ	0.8342	0.9161	0.9161	g Chl a $(mol\ N)^{-1}$	Maximum chlorophyll a to nitrogen ratio	OP
pom_{PHY}	0.0247	0.0269	0.0269	$(mmol\ C\ m^{-3})^{-1}\ d^{-1}$	POM production rate by PHY	OP
r_{SDOM}	0.0068	0.0044	0.0044		parameter for SDOM's lability	OP

μ_{BA}	1.11886	1.9271	1.9271	d^{-1}	Maximum BA growth rate	OP
$resp^{A}_{\max,BA}$	0.7141	0.5468	0.5468	d^{-1}	Maximum BA growth rate	OP
$mort_{BA}$	0.031	0.195	-	d^{-1}	Bacteria mortality rate	
$r_{INFE,BA}$	2.17×10^{-14}	-	2.17×10^{-14}	m^{3} $particle^{-1}$ d^{-1}	Viral infection rate on heterotrophic bacteria	
bs_{BA}	23	-	23		Burst size	
d_{VA}	5.11×10^{-14}	-	5.11×10^{-14}	m^{3} $particle^{-1}$ d^{-1}	Viral decay rate	
μ_{PRT}	1.0938	1.0957	1.0957	d^{-1}	Protozoa (PRT) maximum growth rate	OP
g_{PHY}	3.0012	2.9104	2.9104	mmol C m^{-3}	Half-saturate density of PHY in PRT grazing function	OP
g_{BA}	1.9570	1.5597	1.5597	mmol C m^{-3}	Half-saturate density of BA in PRT grazing function	OP
$resp^{A}_{PRT}$	0.3763	0.3582	0.3582		PRT basal respiration rate	OP
μ_{MZ}	1.1277	1.0039	1.0039	d^{-1}	Maximum metazoa (MZ) growth rate	OP
g_{PRT}	1.4196	1.1410	1.1410	mmol C m^{-3}	Half-saturate density of TR in MZ grazing function	OP
$diss$	0.1215	0.1214	0.1214	d^{-1}	POM dissolution rate	OP

Following parameters values are defaulted from Luo, *et al.* [1]

Parameter	Default value	Unit	Description
A_E	4000		Arrhenius parameter for temperature function
β_{PHY}	0.0002	$(W\ m^{-2})^{-1}$	Light inhibition parameter for photosynthesis of PHY
$v^{N}_{REF,PHY}$	0.3	mol N (mol C)$^{-1}$ d^{-1}	Maximum nitrogen assimilation rate per PHY carbon biomass
k^{NH4}_{PHY}	0.25	mmol m^{-3}	Ammonium half-saturation concentration for PHY growth
k^{NO3}_{PHY}	1.25	mmol m^{-3}	Nitrate half-saturation concentration for PHY growth

$v^{P}_{REF,PHY}$	0.0058	mol P (mol C)$^{-1}$ d^{-1}	Maximum phosphorus assimilation rate per PHY carbon biomass
k^{PO4}_{PHY}	0.06	mmol m^{-3}	Phosphate half-saturation concentration for PHY growth
ζ^{NO3}	2.0	mol C (mol N)$^{-1}$	Carbon requirement (respiration) to assimilate nitrate
$ex_{PSV,PHY}$	0.05	d^{-1}	PHY passive excretion rate (per biomass)
$ex_{CHO,PHY}$	0.05		PHY carbon hydrate excretion rate (per growth rate)
k_{DOM}	0.5	mmol C m^{-3}	Half-saturation concentration of available DOC for heterotrophic bacteria (BA) uptake
b_{RESP}	0.28	(mmol C m^{-3} d^{-1})$^{-1}$	Parameter control BA active respiration rate versus production
$ex_{ADJ,BA}$	2.0	d^{-1}	Bacteria extra semilabile DOC excretion rate
$remi_{BA}$	6.0	d^{-1}	Bacteria inorganic nutrients regeneration rate
$ex_{REFR,BA}$	0.031		Bacteria refractory DOC production rate
$f_{SLCT,BA}$	0.25		BA selection strength on SDOM
$resp^{B}_{BA}$	0.01	d^{-1}	Bacterial basal respiration rate
$resp^{A}_{\min,BA}$	0.35	d^{-1}	Bacteria minimum active respiration rate
ex_{PRT}	0.2		Total DOM excretion rate per PRT gross growth
$f_{ex,PRT}$	0.9		Fraction of labile DOC of total PRT DOC excretion
$resp^{B}_{PRT}$	0.01	d^{-1}	PRT basal respiration rate
$ex_{ADJ,PRT}$	2.0	d^{-1}	PRT extra SDOM excretion rate
$remi_{PRT}$	4.0	d^{-1}	PRT inorganic nutrients regeneration rate
pom_{PRT}	0.028		POM production rate per PRT gross growth
ex_{MZ}	0.2		Total DOM excretion rate per MZ gross growth
$f_{ex,MZ}$	0.9		Fraction of labile DOC of total MZ DOC excretion

$resp_{MZ}^{B}$	0.03	d^{-1}	MZ basal respiration
$resp_{MZ}^{A}$	0.25		MZ active respiration
$ex_{ADJ,MZ}$	2.0	d^{-1}	MZ extra semi labile DOM excretion rate
$remi_{MZ}$	4.0	d^{-1}	MZ inorganic nutrients regeneration rate
pom_{MZ}	0.12		POM production rate per MZ gross growth
$remv_{MZ}$	0.38	(mmol C $m^{-3})^{-1}$ d^{-1}	MZ removal rate by higher-trophic levels
$f_{SDOM,HZ}$	0.14		Fraction of SDOM production by higher-trophic level ingestion
$f_{POM,HZ}$	0.094		Fraction of POM production by higher-trophic level ingestion
$q_{N,REFR}^{C}$	0.05	mol N (mol $C)^{-1}$	Refractory DOM N/C ratio
$q_{P,REFR}^{C}$	0.0007	mol P (mol $C)^{-1}$	Refractory DOM P/C ratio
$q_{N,POM}^{C}$	0.12	mol N (mol $C)^{-1}$	N/C ratio for POM generation by PRT and MZ
$q_{P,POM}^{C}$	0.01	mol P (mol $C)^{-1}$	P/C ratio for POM generation by PRT and MZ
r_{ntrf}	0.15	d^{-1}	Nitrification rate (NH4 to NO3)
prf_{N}	1.05		Preference parameter for dissolving N content in POM
prf_{P}	1.1		Preference parameter for dissolving P content in POM
$wnsv$	30	m	Detritus sinking velocity

Reference

1. Luo, Y.W.; Friedrichs, M.A.M.; Doney, S.C.; Church, M.J.; Ducklow, H.W. Oceanic heterotrophic bacterial nutrition by semilabile DOM as revealed by data assimilative modeling. *Aquatic Microbial Ecology* **2010**, *60*, 273-287, doi:10.3354/ame01427.
